# Genome Sequence of Citrobacter freundii AMC0703, Isolated from the Intestinal Lumen of an 11-Year-Old Organ Donor

**DOI:** 10.1128/MRA.00994-20

**Published:** 2020-11-12

**Authors:** Alan J. Marsh, Kshipra Chandrashekhar, Sandy Ng, Jeff Roach, Scott T. Magness, M. Andrea Azcarate-Peril

**Affiliations:** a Department of Medicine, Division of Gastroenterology and Hepatology, School of Medicine, University of North Carolina, Chapel Hill, North Carolina, USA; b UNC Microbiome Core, Center for Gastrointestinal Biology and Disease, School of Medicine, University of North Carolina, Chapel Hill, North Carolina, USA; c UNC Information Technology Services and Research Computing, University of North Carolina, Chapel Hill, North Carolina, USA; d UNC/NC State University Joint Department of Biomedical Engineering, Chapel Hill, North Carolina, USA; e Department of Cell Biology & Physiology, School of Medicine, University of North Carolina, Chapel Hill, North Carolina, USA; University of Maryland School of Medicine

## Abstract

Citrobacter freundii AMC0703 was isolated from the intestinal mucosa of an 11-year-old organ donor. Genome analysis revealed the presence of multiple factors potentially aiding in pathogenicity, including fimbriae, flagella, and genes encoding resistance to fluoroquinolones, cephamycin, fosfomycin, and aminocoumarin.

## ANNOUNCEMENT

Citrobacter freundii is a Gram-negative, facultative anaerobic bacterium that has been isolated from a variety of environments, including soil and water. C. freundii is also considered to be a commensal organism of the human gut microbiota and an opportunistic pathogen with the ability to cause infection in the bloodstream, urinary tract, and respiratory tract (1). The species is also known to harbor resistance to multiple antibiotics (2).

C. freundii AMC0703 was isolated from the luminal contents of the ascending colon of an 11-year-old female organ donor. Dilutions were plated on an oxygen-reduced rich medium agar and grown in an anaerobic chamber, after which colonies were isolated. The rich medium was composed of glucose (15 g/liter), yeast extract (10 g/liter), proteose peptone (5 g/liter), beef extract (2.5 g/liter), 1.0 ml of MgSO_4_ solution (50 mg/ml), NH_4_H_2_PO_4_ (0.50 g/liter), and 10 ml of hemin (0.5 mg/100 ml). Hemin and vitamin K were added poststerilization. The strain was cultivated in oxygen-reduced rich medium broth, and genomic DNA was isolated (3) and sequenced using Thermo Fisher Ion GeneStudio S5. Raw single end reads were trimmed and processed using BBDuk v38.75 (https://jgi.doe.gov/data-and-tools/bbtools/). A total of 5,748,209 reads were obtained, with an average length of 195 bp. Genomes were assembled using SPAdes (3.14.0) (4) and assessed for completeness and contamination using CheckM (v1.1.2) (5). Annotation was performed using the NCBI Prokaryotic Genome Annotation Pipeline (v4.12) (6). Default parameters were used for all software unless otherwise specified. EzBioCloud was used to determine the average nucleotide identity (ANI) of the strains (7).

**FIG 1:**
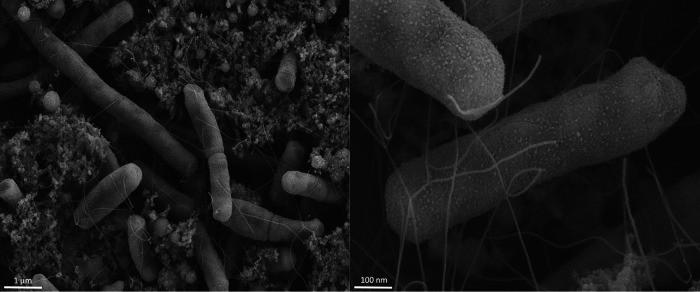
Scanning electron microscope (SEM) imaging of Citrobacter freundii AMC0703. Briefly, bacterial cell pellets were resuspended in 2% paraformaldehyde/2.5% glutaraldehyde in 0.15 M sodium phosphate buffer, pH 7.4. Following treatment as described previously (14), the fixed cell suspension was deposited onto 12-mm round poly-d-lysine-coated coverslips, and following preparation, these were mounted on 13-mm aluminum stubs and sputter coated with 5 nm of a gold-palladium alloy. A Zeiss Supra 25 field emission SEM operating at 5 kV was used to view the AMC0703 isolate with scanning electron microscopy.

AMC0703 has a genome size of 5,057,711 bp across 70 contigs, with 335× genome coverage and an *N*_50_ value of 165,023 bp. Following annotation, there were 4,998 predicted coding sequences and 76 RNA genes. The average GC content is 51.7%. AMC0703 shares 98.77% ANI to the type strain C. freundii ATCC 8090. Phaster (8) identified two intact bacteriophages in the genome homologous to the enterobacterial lambdoid phage mEp213 (44.4 kb) (9) and Haemophilus influenzae phage HP1 (23.8 kb) (10). No CRISPR-Cas genes were found. A number of putative adhesion factors were identified, including genes for a fimbria cluster (Fig. 1) and a type IV pilus. C. freundii is known to be motile, reflected in the genome of AMC0703 by the presence of genes for polar and lateral flagella. Isolates were viewed with scanning electron microscopy (SEM) to visualize these features (Fig. 1). Additionally, the strain contains putative genes for exopolysaccharide and biofilm production. When cultured on medium containing 5% sheep blood agar, the strain displayed hemolytic activity. Among the genes for carbohydrate metabolism were those for lactose, galactose, and maltose, as well as a locus for glycerol uptake and utilization (11). Genes were also present for butanol biosynthesis and for the production of the short-chain fatty acid butyrate. Such functionality remains to be verified experimentally.

The Comprehensive Antibiotic Resistance Database (CARD) (v3.0.7) (9) revealed a large number of genes (28 with >90% identity) for antibiotic inactivation, alteration, and efflux, in particular for fluoroquinolones but also for cephamycin, fosfomycin, and aminocoumarin. Antibiotic resistance is common in C. freundii, with rates likely increasing over time due to overuse of antibiotic compounds (12). The genome also contained regions putatively encoding carocin D and bottromycin, two ribosomally synthesized antimicrobial peptides (13).

### Data availability.

This whole-genome shotgun project has been deposited at DDBJ/ENA/GenBank and SRA under the accession numbers JACCKT000000000 and SRR12606938, respectively. Additional information can be found at the AMC Culture Collection (https://redcap.unc.edu/solutions/microbiome_core_986.php).
